# Biomethane Production from Anaerobic Co-Digestion of Selected Organic Fraction of Municipal Solid Waste (OFMSW) with Sewage Sludge: Effect of the Inoculum to Substrate Ratio (ISR) and Mixture Composition on Process Performances

**DOI:** 10.3390/ijerph182413048

**Published:** 2021-12-10

**Authors:** Santo Fabio Corsino, Michele Torregrossa, Gaspare Viviani

**Affiliations:** Department of Engineering, Università di Palermo, 90128 Palermo, Italy; michele.torregrossa@unipa.it (M.T.); gaspare.viviani@unipa.it (G.V.)

**Keywords:** anaerobic co-digestion, BMP, methane, OFMSW, sewage sludge, synergy

## Abstract

The aim of this study was to evaluate the effect of the inoculum to substrate ratio (ISR) and the mixture ratio between organic fraction of municipal solid waste (OFMSW) and sewage sludge (SS) on the methane production potential achievable from anaerobic co-digestion (AcoD). Biochemical Methane Potential (BMP) assays at mesophilic temperature were used to determine the best AcoD configuration for maximizing methane yield and production rate, as well as to address possible synergistic effects. The maximum methane yield was observed at ISR of 1 and 60% OFMSW: 40% SS as co-digestion mixture, whereas the highest methane production rate was achieved at ISR of 2 with the same mixture ratio (207 mL/gVS/d). Synergistic effects were highlighted in the mixtures having OFMSW below 60%, determining an increase of approximately 40% in methane production than the OFMSW and SS digestion as a sole substrate. The experimental data demonstrated that co-digestion of OFMSW and SS resulted in an increase in the productivity of methane than anaerobic digestion using the sole substrates, producing higher yields or production rates while depending on the ISR and the mixture ratio.

## 1. Introduction

In the last decade, the population growth, the increase of urbanization and the economic development contributed to an overproduction of the municipal solid waste (MSW), which generation is approximately 1.2 kg per capita per day and is expected to increase in the coming decade by more than 50% [1]. The overproduction of MSW led to environmental problems involving air and water pollution, as well as management concerns linked to the high costs and the lack of understanding over different factors that affect the entire management system [2,3]. The organic fraction of municipal solid waste (OFMSW) accounted for approximately 45% (*w/w*) of the MSW and it is considered the main source of environmental impact in landfilling [4]. Indeed, the OFMSW is characterized by a massive amount of putrescible materials because of the presence of residual food waste, kitchen, and restaurant that involve the generation of methane and leachate [5]. To mitigate the environmental pressure caused by the disposal of OFMSW in landfill, alternative management practices have been implemented in recent years, consisting of anaerobic digestion (AD) and/or composting [6]. Anaerobic digestion is a biological process that involves the conversion of organic matter in a mixture of methane (45–55%), carbon dioxide (35–40%) and minor gases (<10%) in different percentages, called biogas. 

At the same time, the worldwide increase of the wastewater treatment plants (WWTP) coupled to even more severe regulations on the discharge limits led to a significant increase in the amount of sewage sludge (SS) to be disposed [7]. Anaerobic digestion is often implemented in large WWTPs to produce biogas for heating and for electricity generation. Furthermore, AD reduces the amount of sludge to be disposed, stabilizes the sludge, destroys the pathogens and limits odour emissions [8]. However, the anaerobic digesters at WWTP are often under-loaded or oversized, leading to under-performing processes and low methane yields and production rates [9]. Several studies explored the possibility to use the free treatment capacity of digesters facilities in WWTP for the anaerobic co-digestion (AcoD) of SS with OFMSW [8,9]. AcoD has numerous benefits such us improving moisture content, adding micro and macro nutrients and leading to more appropriate C:N ratios [1]. Beside the benefit of boosting the biogas production, it was demonstrated that the AcoD enabled to accelerate the digestion process, to increase biogas yield and to lead higher degradation rates [9]. Moreover, inhibitory compounds are diluted due to blending and often beneficial synergetic effects could be achieved in co-digestion unlike in mono-digestion [10]. Therefore, the AcoD is a suitable route for producing methane resulting in a source of renewable energy required for a successful transition of WWTP to biorefinery concept.

However, researchers have obtained contradictory results on this topic, thereby suggesting that optimization of the substrate mix ratio is required for achieving stable operation and high methane yield. Indeed, Kim et al. [11] found that the optimum mixture ratio for OFMSW and SS was 50% of volatile solids (VS) for both substrates, whereas Nielfa et al. [10] obtained the highest methane yield with 80% of OFMSW and 20% of SS. Similarly, Jansen et al. [12] defined an optimum of 80% VS for SS and 20% for OFMSW, whereas Bjorn et al. [13] obtained their best results with a mixture corresponding a 3:1 ratio between OFMSW and SS on VS basis. To estimate the optimum ratios between co-substrates when co-digestion is intended, specific preliminary assays should be performed. In this sense, biochemical methane potential (BMP) assays are applicable to understand accurately the properties of the substrate to be treated. 

In addition to the composition of the substrate mixture, in previous literature it was demonstrated that the methane yield and production rate are affected by the ratio between the inoculum and the substrate (ISR) [14]. ISR is considered as the most important factor for improving high methane yield and digester stability [15]. Generally, larger inoculation doses in AD shorten the start-up time and increase the specific methane production rate by providing greater buffering capacity and more methanogens [16]. However, excessive inoculum requires more space and decreases the volumetric methane production rate. In contrast, very low inoculum doses can induce AD failure. According to Motte et al. [17], the S/I ratio effects only the start-up phase, TS content is the main parameter governing methane production during the growing phase of AD. Slimane et al. [18] observed that the biogas production increased as the ISR value decreased (<1), whereas Raposo et al. [14] suggested that the ISR should be higher than 2 to avoid inhibitory effects. The ISR is a crucial operating parameter in AcoD since it is related to the volume of the anaerobic digesters or to their free treatment capacity [19]. 

To the Authors’ best knowledge, an aspect that has not yet been addressed in the literature is the combined effect of the ratio of substrates in the co-digestion mixture and the ISR on the methane yield and production rate obtainable from the AcoD between the OFMSW and SS.

In light of this, the objective of this study was to evaluate the effects of the ISR and the mixture proportion of sewage sludge with OFMSW on methane production potential achievable from anaerobic co-digestion of these two substrates to optimize the above operational parameters for further experiment in continuous operating digesters. More precisely, BMP assays with ISR equal to 0.05, 0.5, 1, 2, and mixture ratio ranging from 20% to 80% were carried out to assess the maximum methane yields and kinetics.

## 2. Materials and Methods

### 2.1. Organic Waste, Sewage Sludge and Inoculum

Synthetic OFMSW was produced in laboratory, in order to make easier the comparison between the results of different tests. The different fractions of the OFMSW were chosen in order to best represent the organic fraction of urban waste from door-to-door separate collection, according to the average composition of the OFMSW in Italy [4]. More precisely, the OFMSW was obtained by mixing residues of pasta (10% wt), bread (10% wt), vegetables (45% wt), fruits (25%) and meat/cheese (10% wt). The OFMSW samples were shredded to obtain an average particle size of 20 mm. 

The sewage sludge was collected from a pre-thickened unit of a municipal WWTP located in Palermo (Italy). The sewage sludge was a mixture of primary sludge and activated sludge from the biological unit, characterized by a total solids (TS) content approximately of 2% TS. The sludge was sieved through a 5 mm mesh-sieve to remove coarse particles before each BMP test. 

The inoculum was collected from a bench scale anaerobic digester operating under mesophilic conditions (T = 35 °C) that was fed with the thickened sludge collected from the above mentioned WWTP and a mixture of acetate and trace elements to enhance the growth of methanogenic bacteria [20]. The TS content of the inoculum was close to 1.8% on average, whereas the ratio between VS and TS was approximately 0.67. 

### 2.2. Biochemical Methane Potential Assays

BMP assays were performed in four experimental phases. In each phase, the BMP assays were conducted at different ISR equal to 0.05, 0.5, 1, 2 based on the volatile solid (VS) content. Moreover, for each ISR six different mixture between OFMSW and SS were evaluated. In all the samples, the percentage of total solids was maintained below 5% TS, to simulate a wet anaerobic digestion process. Glass bottles with a working volume of 500 mL were used for all the assays. The volume of the mixture between OFMSW, SS and inoculum added in each bottle was 300 mL, thus the headspace volume was of 200 mL. Before starting the anaerobic digestion, each bottle was fluxed by nitrogen gas. In all the tests, no pH adjustment was performed because of the high buffer capacity of the sewage sludge and to assess the process behavior without any chemical addition [21]. Then, bottles were sealed and connected to a Tedlar bag in which the biogas produced was collected. The bottles were placed within a thermostatic chamber under controlled temperature (35 °C) on a magnetic plate that ensured their mixing. Hereafter, every 2–3 days, the volume of methane accumulated within the bag was measured through a liquid-displacement method, using an alkaline solution (2% NaOH) as barrier-liquid. The BMP assays were finished when a daily production of less than 1% of the entire production occurred. The results provided by the BMP assays were expressed as the volume of methane per gram of VS added (mL/gVS_added_). 

### 2.3. Co-Digestion Mixtures

Six different co-digested mixtures including selected OFMSW and SS were considered in this study to evaluate the optimum ratio for the co-digestion of these two substrates. More precisely, the percentage of OFMSW and SS was increased from 20% to 80% (+20% in each test). Moreover, to assess the potential synergistic effect of combining OFMSW and SS, two reactors were started using the sole substrates. The same mixtures were replicated for each assay at different ISR (0.05, 0.5, 1, 2). Table 1 summarizes the composition and the concentrations of the main physical-chemical parameter of each mixture.

### 2.4. Calculation

The data of cumulative methane production obtained from BMP assays were interpolated using an exponential equation (Equation (1)) [4]:

(1) $$P\left(t\right)=P_{tot}\cdot \left(1-e^{-k\cdot t}\right)$$

where *P*(*t*) is the methane production at a generic time, *P_tot_* is the cumulative value of methane produced at the end of BMP, *k* is the rate of methane production and *t* is the time. *P_tot_* and *k* were estimated using the solver function of Excel (MS Office), by minimizing the sum square of errors between the experimental data obtained from BMP assays and the results from the model. 

### 2.5. Assessment of Synergistic Effects

Anaerobic co-digestion was supposed to provide advantages over AD with mono-substrate because of the establishment of synergistic effects that increase the biogas production. To assess the mutual influence of the ISR and the different mixtures of OFMSW and SS on methane production, the synergistic effect (*SE*) was calculated by the (Equation (2)):

(2) $$SE=\frac{BMP_{mix-i}}{BMP_{SS,i+OFMSW,i}}$$

where, the *BMP_mix−i_* is the cumulative methane production obtained in each co-digestion mixture (2–5), whereas *BMP_SS,i+OFMSW,i_* represents the theoretical methane production obtainable from the above mixture. The latter was calculated considering the specific methane productivity (mL/gVS_added_) of the sole substrate (SS and OFMSW) obtained from the assays of Mix1 and Mix6, and the VS of each substrate contained in the co-digestion mixtures (2–5).

A value lower than the unit indicated that the mixture had a competitive effect in the final methane production, whereas a higher value indicated that the mixture had a synergistic effect in the final production [10].

### 2.6. Analytical Methods

The samples of the organic solid waste and sewage sludge were analyzed for the content of total solids (TS), volatile solids (VS), chemical oxygen demand (COD), total nitrogen (TN), total phosphorous (TP) and moisture, according to Standard Methods [22]. The content of proteins, carbohydrates and lipids was analyzed according to the methods reported in the literature [23,24,25]. The characterization of each co-digestion mixtures was obtained from the theoretic mixture of the sole OFMSW and SS. 

## 3. Results and Discussion

### 3.1. Reduction of VS

**Figure 1:**
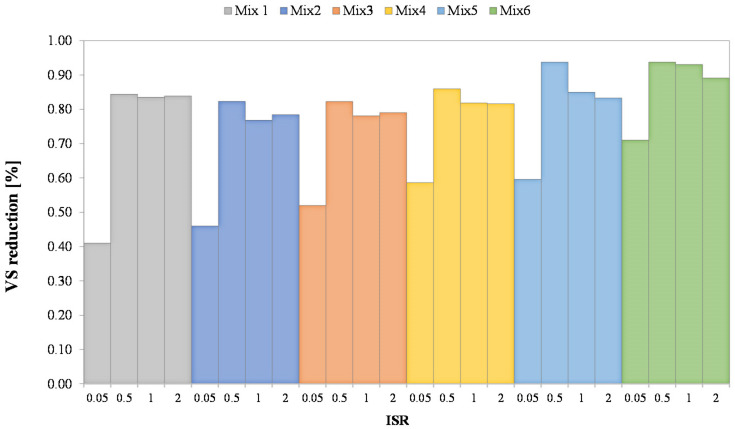
Reduction of VS obtained in the six mixtures of OFMSW and SS at different ISR.

To assess the effectiveness of the anaerobic digestion process, the reduction of VS at the end of each BMP assay was calculated as the percentage difference between the initial and final content of VS within each co-digestion mixture. Figure 1 depicts the VS removal efficiencies obtained with the variation of the ISR and the mixture composition.

The VS reduction obtained at the lowest ISR (0.05) was significantly lower than the other assays. Indeed, the average reduction of VS was close to 55%, while showing an increasing tendency with the percentage of OFMSW in the mixture. The highest VS reduction was obtained in the Mix6 (71%), thereby indicating that the VS reduction performances increased with the initial content of VS in the mixture. The low values of VS reduction obtained at ISR of 0.05 could be attributed to the scarce biodegradability of the activated sludge and to the large volume of solid waste compared to the inoculum that could result in accumulation of ammonia or volatile fatty acids that could inhibit methanogens. Existing reports indicated that a low ISR increases the concentration of fatty acids and hence reduces the pH [26,27]. Furthermore, another study reported that the inhibition of the AD process could occur in reactors containing a high amount of protein-rich material [28]. Indeed, since the SS was characterized by a high content of protein than the OFMSW, it was possible to speculate that in the mixtures with a high percentage of SS (Mix1, Mix2, Mix3) the decomposition of proteins led to an over-accumulation of ammonium that resulted in AD inhibition.

The reduction of VS significantly increased in BMP assays performed at ISR higher than 0.5. The VS removal was higher than 80% in all the mixtures and even in this case an increasing tendency with the percentage of OFMSW in the mixture was noted. The highest VS removal was obtained with the Mix6 (OFMSW only) and resulted close to 92% on average. Therefore, any inhibition was observed at ISR higher than 0.5. Anaerobic digestion involves a series of processes including hydrolysis, acidogenesis (acetogenesis), and methanogenesis. Therefore, a build-up of volatile fatty acids could occur if a kinetic uncoupling between the acid producers and consumers occur [29]. Thus, if the amount of methanogens bacteria is sufficient compared with that of the organic substrate and any other inhibiting factors are present the AD process is not limited. The results indicated that a minimum ISR close to 0.5 is recommended to prevent any inhibition of the AD process, thus ensuring high VS removal efficiency. Moreover, it should be stressed that the above results were significantly higher than that reported in other studies [10,30]. This was likely because the OFMSW used in this study was specifically reproduced in the laboratory and did not contain any impurities that might have had in a “real” OFMSW. Besides, the initial shredding and the small particle size might had significantly improved the AD performances [31]. 

### 3.2. Methane Production Yield

**Figure 2:**
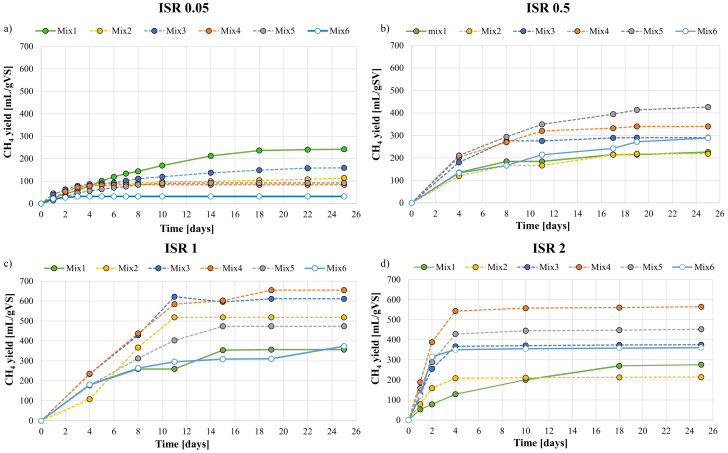
Cumulative methane yields curves of OFMSW and SS at: (**a**) ISR equal to 0.05; (**b**) ISR equal to 0.5; (**c**) ISR equal to 1; (**d**) ISR equal to 2.

BMP assays at different ISR and mixture ratio pointed out that both these parameters significantly influenced the methane yield and production rate. All the assays were run for 25 days, although in many cases the maximum methane yield was achieved in a shorter time. Figure 2 depicts the cumulative trends of methane production in BMP performed at ISR of 0.05 (a), 0.5 (b), 1 (c) and 2 (d). 

At ISR of 0.05, Mix 1 had the best methane yield (242 mL/gVS). Thus, the highest methane production was achieved when the activated sludge as the sole substrate was used. The other co-digestion mixtures produced less methane proportionally with the increase of the OFMSW in the mixture. This result was apparently in contrast with the increase of VS removal observed with the increase of the OFMSW in the mixture. Indeed, a previous study reported that an increase in the VS removal and a reduction of the methane yield were observed at low ISR [32]. In this regard, it is possible that at low ISR, methanogenesis was the limiting step of AD. Indeed, since no limitations in acidogenesis were observed, incremental accumulation of VFA occurred as the initial VS of the mixture increased because of the high availability of VS compared to methanogens bacteria. Therefore, this resulted in a higher removal efficiency of VS as its percentage in the mixtures increased, while reducing the methane yield on the other because VFA accumulation resulted in the inhibition of methanogens bacteria [27].

At ISR of 0.5, the highest methane yield was obtained with the Mix5 (426 mL/gVS). Apart from mix2, all of the mixtures had higher methane productions than the OFMSW and SS as sole substrates, thereby suggesting the occurrence of synergistic effects on AcoD. Similarly, at ISR of 1 and 2, the highest methane production was equal to 655 mL/gVS and 565 mL/gVS, respectively, obtained in both cases with Mix 4. Additionally, in both the BMP assays, co-digestions increased the methane productivity of the OFMSW and SS as sole substrates, thereby suggesting that neither competitive effects nor inhibition in methanogenesis occurred at ISR higher than 0.5. 

**Figure 3:**
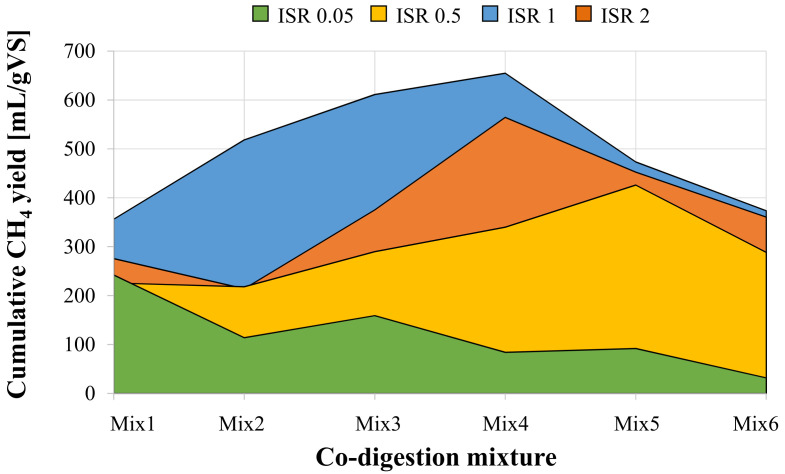
Cumulative methane production obtained in BMP assays at different ISR and mixture ratio.

Figure 3 summarizes the cumulative methane production achieved in each BMP assays as a function of the ISR.

As was previously discussed, the cumulative methane yield decreased with the increase of the OFMSW fraction in the mixture at ISR of 0.05. In the other assays, the cumulative methane production as a function of the OFMSW/SS mixture showed a similar behavior. Specifically, in all the assays carried out at ISR higher than 0.5, the cumulative methane production showed a maximum in corresponding of a specific mixture, thereby indicating the existence of an optimum composition for the co-digestion of OFMSW and SS at a generic ISR. More precisely, the maximum methane productivity (655 mL/gVS) was obtained at ISR 1 with the co-digestion of OFMSW and SS with a percentage of 60% and 40%, respectively. With the same mixture, at ISR 2 the maximum methane production was of 565 mL/gVS, whereas a lower value (426 mL/gVS) was obtained at ISR 0.5 although with an OFSMW fraction of 80%. The above results indicated that the best configuration for co-digestion in terms of methane yield was OFMSW (60%) and SS (40%) with ISR 1. The existence of a maximum value in correspondence of a precise mixture of OFMSW and SS, suggested that the process could be limited for lower and higher values. Indeed, when the SS fraction was prevailing in the mixture, it is possible that the lower C:N, and the higher concentration of ammonium resulting from proteins dissimilation, as well as the higher alkalinity, could result in limitation of the AD process, hence in the methane production [1]. On the other hand, although the increase of OFMSW supplemented additional nutritional components and stabilize alkalinity, an excessive amount of substrate could result in the decrease of biogas yield due to VFA accumulation [33,34]. Therefore, preliminary tests like BMP are necessary when co-digestion is intended to find the optimum mixture of the two substrates, thereby avoiding process limitation and inhibition, as well as ensuring high methane yields. 

### 3.3. Evaluation of Synergistic Effects

**Figure 4:**
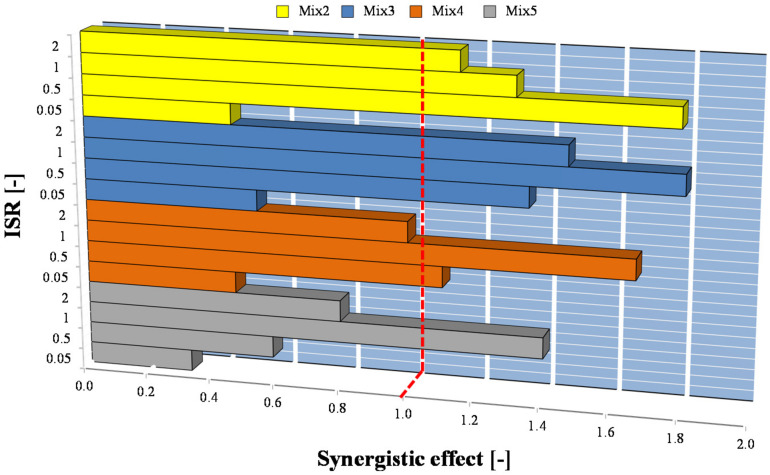
Results of the synergistic effect obtained in co-digestion of OFMSW and SS at different mixtures and ISR.

Previous literature reported that co-digestion of OFMSW and SS can produce synergistic effects involving additional methane yields if a proper balancing between substrates is achieved [9,10]. Nonetheless, lower methane yields are obtained if competitive effects arise. Data obtained in BMP assays highlighted that the methane production obtained with some mixtures was higher than that achieved with the OFMSW and SS as sole substrates. Figure 4 reports the value of the synergistic effect calculated according to Equation (2) in each BMP assay. 

The results shown in Figure 4 suggested that an antagonism effect was observed in all the assays performed at ISR of 0.05. As was previously discussed, very low VS reduction and methane yields were observed in these assays, thereby suggesting the occurrence of limitations and inhibition to the AD process. Considering the assays performed at ISR higher than 0.5, synergistic effects were observed mainly with co-digestion of Mix2 and Mix3, whereas when the OFMSW fraction increased (Mix 4 and Mix 5), synergistic effects were only noted at ISR of 1. The above results indicated that synergistic effects occurred when the percentage of OFMSW in the co-digestion mixture was near 60%, whereas the occurrence of antagonistic effects or any additional improvement in methane yields were observed when the OFMSW increased. These results might explain why the cumulated methane production decreased after a certain amount of OFMSW in the co-digestion mixture (see Figure 3). The results obtained in this study were in contrast with those reported in another study [10], whereas they were consistent with that obtained by Aichinger and co-authors [9]. This difference could be related to a different composition of both the SS and OFMSW that might affect the mutual influence of these two substrates in AcoD process. This strengthens the need to carry out preliminary BMP assays to optimize the mixture ratio of the substrates and the ISR, according to the specific composition of the substrates to be co-digested. 

### 3.4. Kinetics of Methane Production

**Figure 5:**
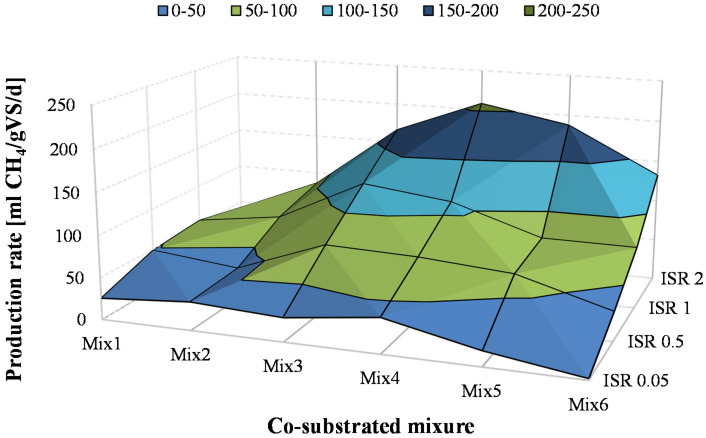
Methane production rates obtained in BMP assays.

The cumulative methane curves reported in Figure 2 indicated that the kinetics of methane production were significantly affected by both the ISR and the composition of co-substrates mixtures. Figure 5 reports the maximum production rate (P_tot_ × k) as a function of the co-substrate mixture and the ISR. 

The lowest rates of methane production were obtained at ISR of 0.05 (<40 mL/gVS/d) and the values decreased as the OFMSW in the co-digested mixture increased. This result confirmed that high OFMSW at low ISR caused inhibition of methanogens, likely due to excessive VFA accumulation. The methane production rates increased with the ISR. Indeed, the highest values, close to 210 mL/gVS/d were obtained at ISR 2, whereas lower values were achieved at ISR 1 (83 mL/gVS/d) and ISR 0.5 (56 mL/gVS/d), on average. Moreover, independently of the ISR, the methane production rates showed a maximum value in correspondence with a specific co-substrate mixture, thereby indicating that the ratio between the OFMSW and SS in the co-digested mixture affected not only the maximum methane yield but also its production rate. Overall, the maximum methane production rate (207 mL/gVS/d) was obtained at ISR 2 in correspondence with a mixture constituted by 60% OFMSW and 40% SS.

The above findings indicated that the operating conditions for the maximum cumulative methane yield (655 mL/gVS at ISR 1 and Mix 4), did not provide the highest methane production rate (109 mL/gVS/d), which was instead achieved at ISR 2 and Mix 4 (207 mL/gVS/d) that produced 565 mL/gVS. Overall, the methane production rates were slightly higher than that reported in other studies on OFMSW with a similar composition [4,10], although the latter were obtained with real OFMSW and without a preliminary shredding that could significantly affect the methane production kinetics. 

### 3.5. General Remarks and Considerations for Continuous Mode Operation

What above discussed indicated that the composition of the co-substrate mixture and ISR affected the kinetic of the AcoD process. According to the literature, the methane yield should be theoretically independent of the ISR, and this only affects the kinetics of the methane production [14,35]. Moreover, it was demonstrated that the use of high inoculum concentration required shorter adaptation time (lag phase), ensuring high methane production rates in the early stage of the AD process. However, data obtained in this study demonstrated that ISR had remarkable influence on both. Indeed, a lower ISR produced a higher synergistic effect, although showing a lower methane production rate. Nevertheless, as reported in other study, the effect of the ISR on methane productivity should be only limited to the start-up phase [17], whereas at steady state its effect is negligible compared to other parameters. Furthermore, it was demonstrated that the use of more inoculum amount in co-digestion process had no significant influence on biogas generation beyond certain point. However, excessive use of inoculum leads to an increase in digester volume unnecessarily required for the co-digestion [19]. 

From an operating point of view, a short lag phase implies the possibility to operate in digesters with a smaller volume, since high methane production rate could be achievable at short time, hence at low hydraulic retention time. Conversely, lower methane production rate requires larger digesters volume that could allow producing higher methane yield. The reason why higher methane yields at low ISR were obtained in the present study, could be due to the higher biodegradability of the OFMSW matrix, since this did not contain impurities being constituted by residual food fraction only. This could involve that a limited VFA accumulation was obtained at low ISR, in contrast with what reported in previous literature [17]. 

## 4. Conclusions

The results obtained in this study demonstrated that the ISR and the ratio OFMSW:SS in the co-digestion mixture significantly affected the methane yield, the production rate and the synergistic effect produced during the biodegradation process. If on the one hand a lower inoculum concentration (ISR 1) produced the highest methane yield (655 mL/gVS) and synergistic effects (+40%), on the other the increase of inoculum (ISR 2) enabled the highest methane production rate (207 mL/gVS/d). Moreover, although low ratios of OFMSW:SS (<40–60%) in the co-digestion mixture resulted in the highest synergistic effects, as long as the ISR was higher than 0.5, they produced lower methane yields and production rates. Similarly, high OFMSW (<80–20%) resulted in a decrease of both the methane yields and production rates likely due to process inhibition. Overall, referring to the composition of the co-digestion mixture an optimum mixture ratio close to 60% OFMSW and 40% SS was found, whereas the ISR produced conflicting results. Nonetheless, the results obtained in BMP assays should be validated in continuous-mode operation anaerobic digesters. 

## Figures and Tables

**Table 1:** Ratio of mixtures and composition for BMP.

Mixure ID	OFMSW/SS	Percentage TS	Moisture	VS	TN	TP	COD	PN	CS	LS
–	%dw	%TS	%	%TVS	mgN/gTS	mgP/gTS	mgO_2_/gTS	%TS	%TS	%TS
**ISR = 0.05**										
Mix 1	0–100	2.82	97.2	72%	6.3	1.98	963.57	75.29	22.40	0.21
Mix 2	20–80	3.30	96.7	80%	10.9	1.85	1184.55	57.73	32.34	6.51
Mix 3	40–60	4.88	95.1	82%	12.4	1.90	1256.62	51.83	35.39	9.08
Mix 4	60–40	5.07	94.9	87%	15.3	1.87	1389.61	41.36	41.53	13.64
Mix 5	80–20	5.04	95.0	91%	18.2	1.83	1530.44	29.63	47.91	18.83
Mix 6	100–0	5.81	94.2	95%	20.8	1.96	1644.32	17.67	55.06	23.51
**ISR = 0.5**										
Mix 1	0–100	1.51	98.5	74%	6.4	4.33	944.94	63.41	33.21	0.78
Mix 2	20–80	1.60	98.4	76%	7.8	4.46	1005.77	59.62	36.13	1.61
Mix 3	40–60	1.72	98.3	77%	9.3	4.60	1071.97	54.92	39.12	3.36
Mix 4	60–40	1.86	98.1	79%	10.9	4.79	1144.22	49.66	42.43	5.29
Mix 5	80–20	2.05	98.0	81%	12.8	4.97	1223.82	43.84	46.18	7.34
Mix 6	100–0	2.31	97.7	84%	14.8	5.17	1311.62	37.47	50.23	9.73
**ISR = 1**										
Mix 1	0–100	1.34	98.7	72%	6.4	5.67	917.85	59.44	37.51	0.51
Mix 2	20–80	1.39	98.6	74%	7.4	5.81	961.80	56.53	39.53	1.57
Mix 3	40–60	1.44	98.6	75%	8.5	5.94	1008.67	53.41	41.54	2.63
Mix 4	60–40	1.50	98.5	76%	9.7	6.13	1058.02	50.33	43.88	3.71
Mix 5	80–20	1.57	98.4	77%	10.9	6.30	1111.29	46.41	46.23	5.06
Mix 6	100–0	1.66	98.3	79%	12.2	6.50	1167.01	42.67	48.71	6.21
**ISR = 2**										
Mix 1	0–100	1.51	98.5	70%	5.2	5.58	719.89	55.96	40.66	0.73
Mix 2	20–80	1.54	98.5	71%	5.7	5.67	738.02	54.01	41.92	1.32
Mix 3	40–60	1.58	98.4	71%	6.2	5.75	758.10	52.11	43.22	1.94
Mix 4	60–40	1.61	98.4	72%	6.8	5.81	780.17	50.16	44.43	2.66
Mix 5	80–20	1.65	98.3	72%	7.3	5.90	801.54	48.06	45.98	3.31
Mix 6	100–0	1.69	98.3	73%	7.9	6.01	822.52	45.83	47.44	4.12

Table legend: OFMSW/SS: ratio between organic fraction of municipal solid waste (OFMSW) and sewage sludge (SS); total solids (TS); volatile solids (VS); TN: total nitrogen; TP: total phosphorous; COD: chemical oxygen demand; PN: proteins; CS: carbohydrates; LS: lipids.

## Data Availability

Data will be available on request to the corresponding author.
